# Effect of exercise-based rehabilitation on functional capacity and renal function in type 2 diabetes mellitus with nephropathy: a randomized controlled trial

**DOI:** 10.1007/s11255-024-03987-w

**Published:** 2024-03-14

**Authors:** Megha Nataraj, G. Arun Maiya, Shankar Prasad Nagaraju, B. A. Shastry, K. N. Shivashankara, Sahana Shetty, Shreemathi S. Mayya

**Affiliations:** 1 Department of Physiotherapy, Centre for Podiatry and Diabetic Foot Care and Research (CPDFCR), Manipal College of Health Professions (MCHP), Manipal Academy of Higher Education (MAHE), Manipal, Karnataka 576104 India; 2 Department of Nephrology, Kasturba Medical College (KMC)-Manipal, Manipal Academy of Higher Education (MAHE), Manipal, Karnataka 576104 India; 3 Department of Medicine, Kasturba Medical College (KMC)-Manipal, Manipal Academy of Higher Education (MAHE), Manipal, Karnataka 576104 India; 4 Department of Endocrinology, Kasturba Medical College (KMC)-Manipal, Manipal Academy of Higher Education (MAHE), Manipal, Karnataka 576104 India; 5 Department of Data Science, Prasanna School of Public Health (PSPH), Manipal Academy of Higher Education (MAHE), Manipal, Karnataka 576104 India

**Keywords:** Diabetes, Exercise, Nephropathy, Functional capacity, Rehabilitation

## Abstract

**Introduction:**

Diabetic nephropathy is a growing public health challenge with implications on health. Renal function decline impacts the functional ability and overall health and well-being of individuals with diabetic nephropathy due to development of several renal manifestations. The objective of the study was to determine the effect of an exercise-based rehabilitation program on functional capacity and renal function among individuals with type 2 diabetic nephropathy.

**Methods:**

A total of 283 individuals were screened and 60 eligible participants aged 45–70 years with diabetic nephropathy were randomly allocated (*n* = 30 each) to the intervention group (IG) and control group (CG), respectively. The study outcome measures comprised of functional capacity (6-min walk test) and renal function assessed at baseline, 12th week and 24th week. Participants allocated to IG received 12 weeks of exercise based rehabilitation (comprising of supervised + home-based exercises) along with standard care and followed-up till 24th week.

**Results:**

The repeated measures ANOVA with Greenhouse–Geisser correction indicated significant timepoint*group interaction effect for 6-min walk distance F _(1.71, 90.59)_ = 619, *p* < 0.001, serum creatinine F _(1.23, 65.14)_ = 174.8, *p* < 0.001, estimated glomerular filtration rate *F*_(1.15, 60.88)_ = 105.2, *p* < 0.001, serum urea *F*_(1.48, 78.45)_ = 261.4, *p* < 0.001 and urine protein F _(1.13, 59.82)_ = 4.58, *p* < 0.328.

**Conclusion:**

The study found that exercise based rehabilitation improved both functional capacity and renal function among individuals with type 2 diabetic nephropathy.

## Introduction

Diabetes mellitus (DM) is a major cause for chronic kidney disease (CKD) worldwide [1] with several health implications. Diabetic nephropathy (DN) results in structural and functional changes within the nephron causing kidney function deterioration [2]. Diabetes-induced chronic kidney disease has contributed to an increase in the disability adjusted life years (DALYs) [3, 4]. The untimely recognition of kidney dysfunction acts as a major contributor for the progression of diabetic nephropathy to late stages as end-stage renal disease (ESRD) or chronic renal failure (CRF) demanding immediate management through haemodialysis (HD) /peritoneal dialysis (PD) or renal replacement therapy (RRT) [5].

The early stages of diabetic nephropathy (stage 1, 2, 3A) remain asymptomatic and underdiagnosed among individuals while late stages (stage 3B, 4, 5) represent renal manifestations: fatigue, sarcopenia, anaemia, reduced physical function and quality of life [6, 7]. During the late stages of renal dysfunction, there is a restricted risk interval period among individuals. Thus, strategies aimed largely at early identification and screening for kidney dysfunction among individuals with diabetes not only aid in the delivery of optimal management but also serve as a strong focus of clinical nephrology research [8]. They help to address the sustainable development goal-3 on good health and well-being by preventing the progression to end-stage renal disease (ESRD)/chronic renal failure (CRF) with minimal socio-economic burden [9, 10].

Exercise and lifestyle modification produce numerous health benefits regardless of the age of an individual. Several studies have proven the beneficial role of exercise on cardiorespiratory endurance, inflammatory markers, among individuals with type 2 diabetes mellitus and chronic kidney disease (CKD) individually [11]. However, despite a high global prevalence and disease progression of diabetic nephropathy, the role of an exercise-based rehabilitation program for functional capacity and renal function have not been studied. Thus, the objective of the present study was to determine the effect of exercise based rehabilitation on functional capacity and renal function for type 2 diabetes mellitus individuals with nephropathy.

## Methodology

### Study design

Randomized controlled trial (RCT).

### Study setting

Centre for Podiatry and Diabetic Foot Care & Research, Outpatient units of Department of Nephrology, Medicine & Endocrinology at Kasturba Hospital, Manipal, Karnataka, India.

### Ethical approval

The study was approved by the Institutional Ethics Committee [IEC-329/2020] and thereafter registered under the Clinical Trail Registry of India [CTRI/2020/09/027677].

### Sample size

The calculated sample size for the study was 19 participants in each group for *α* = 0.05, 80% power, *σ* = 2.1, *d* = 1.5, three timepoints, *ρ* = 0.4. Accounting for 30% drop rate, the same size was 27 per group. Thus, rounding of to 30 per group, the total sample size for this randomized controlled trial was 60 participants.

### Study eligibility criteria

Individuals with physicians diagnosed type 2 diabetes mellitus with nephropathy, in age group 45 to 70 years, of any gender, cognitively stable to provide consent for participation in the study were included. Individuals diagnosed with type 1 diabetes mellitus or type 2 diabetes mellitus with stage 5 chronic kidney disease who have been initiated haemodialysis/maintenance dialysis/peritoneal dialysis/renal transplant/renal replacement therapy with an estimated glomerular filtration rate below 15 ml/min/1.73m^2^ were excluded. Those who have undergone any recent cardiovascular event/procedure in last 6 months, or presence of any neuromuscular condition/disease that limits their ability for exercise testing and prescription were excluded.

### Screening of participants

**Fig. 1:**
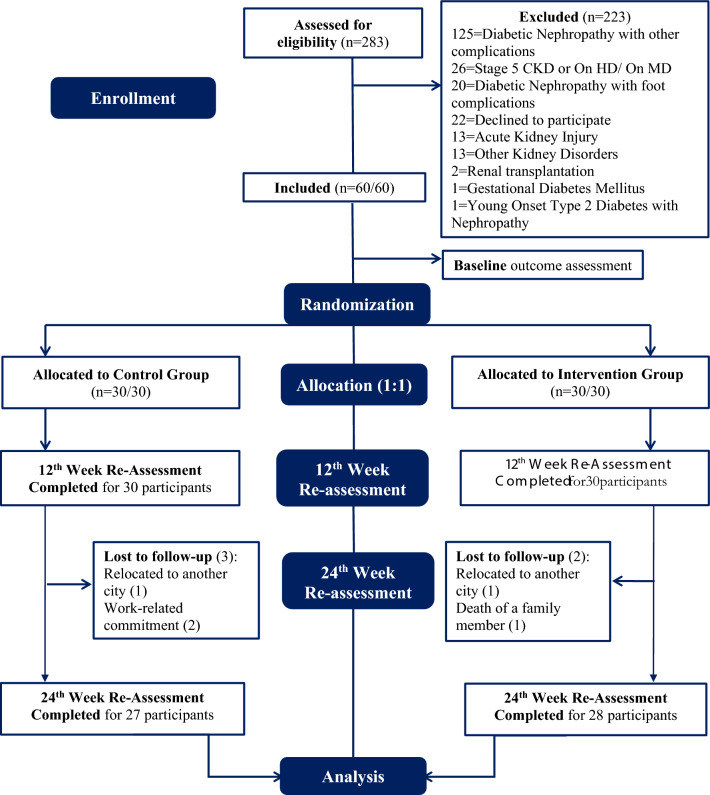
Consort flow diagram

Individuals were screened between December 2020 to December 2022 at the Centre for Podiatry and Diabetic Foot Care & Research, Outpatient units of Department of Nephrology, Medicine & Endocrinology at Kasturba Hospital, Manipal, Karnataka, India. The details of the screening procedure are provided in Fig. 1**.**

### Randomization, allocation and blinding

All participants were assessed for their eligibility. Those meeting the study criteria were explained about the study procedure and were recruited after they provided their written informed consent for study participation. At baseline, their detailed demographic data were recorded along with assessment of study outcome measures of functional capacity using 6-min walk distance and all renal function parameters.

The included participants were thereafter randomly allocated to either the Control Group (CG) or the Intervention Group (IG) with computerized block randomization procedure using a sealed envelope method. A total of ten blocks were generated. Each block contained six participants from both the Control Group (CG) and the Intervention Group (IG), to avoid any selection and allocation bias. All outcome assessments were performed by a qualified physiotherapist who was blinded.

### Study outcome measures

The study outcome measures were assessed at three timepoints: baseline, after completion of 12 weeks and after completion of 24 weeks.

#### Functional capacity

The functional capacity of study participants were assessed using the 6-min walk test (6MWT) in accordance with American Thoracic Society (ATS) guidelines [12]. 6MWT is a submaximal exercise test that is commonly used for assessment of functional capacity among individuals with chronic pulmonary or cardiac conditions. It is a reliable assessment tool and has been widely accepted and utilized in clinical and research settings for chronic disease population groups.

#### Renal function

The biochemical assessments of the study participants were measured in accordance with the standard guidelines of the Biochemistry Laboratory of Kasturba Hospital, Manipal. These included the following: serum creatinine, serum urea nitrogen, glycated hemoglobin, fasting blood glucose, which were performed using venous blood of the participant drawn after 8 to 10 h of fasting state. However, the post-prandial blood glucose was measured after 2 h of food intake of the participants. The urine sample (morning-fasting state) of participants were collected to assess the urine protein level. Serum creatinine (mg/dL) was measured using the standardized Jaffe method [13]. The normative values are as follows: male [0.7 to 1.2 mg/dL] & female [0.5 to 0.9 mg/dL]. Serum urea-nitrogen (mg/dL) was measured using Urease Glutamate dehydrogenase (GLDH) method [14]. The normative values range between 10 and 40 mg/dL. Urine protein level was measured using the protein error of pH indicators method (mg/dL) [15]. The urine protein values were categorized as follows: Negative urine protein test, Trace [≤ 15 mg/dL], 1 + [30 mg/dL], 2 + [100 mg/dL], 3 + [300 mg/dL] and 4 + [≥ 1000 mg/dL]. Estimated glomerular filtration rate (mL/min/1.73m^2^) was calculated using the Chronic Kidney Disease Epidemiology Collaboration (CKD-EPI) creatinine-based equation [16] with categorization into stages as follows: Stage 1[≥ 90 mL/min/1.73m^2^], Stage 2[60 to 89 mL/min/1.73m^2^], Stage 3A [45 to 59 mL/min/1.73m^2^], Stage 3B [30 to 44 mL/min/1.73m^2^] and Stage 4 [15 to 29 mL/min/1.73m^2^].

### Study intervention

All study participants received the following interventions regardless of their allocated groups.

#### Standard physician care

Each participant received standard physician care from the consulting doctor at the Kasturba Hospital at baseline, 12th week and 24th week.

#### Patient education and counselling

Every participant received the detailed patient education on type 2 diabetes mellitus, its implications on kidney function, importance of maintaining optimal kidney health, incorporating lifestyle modification (through diabetic-kidney specific diet and exercises). Participants & their caregivers were encouraged to discuss their concerns and queries with the study investigator.

#### Diet and nutritional plan

Each participant received an individualized diet and nutrition plan from a senior dietician at the Department of Clinical Dietetics & Nutrition of Kasturba Hospital.

However, the study participants allocated to intervention group received a detailed exercise based rehabilitation programme for a period of 12 weeks (i.e., exercise phase). It comprised of both supervised and home-based exercise sessions. After completion of exercise phase, these participants were followed up till completion of 24 weeks (i.e., maintenance phase).

#### Exercise-based rehabilitation program

**Fig. 2:**
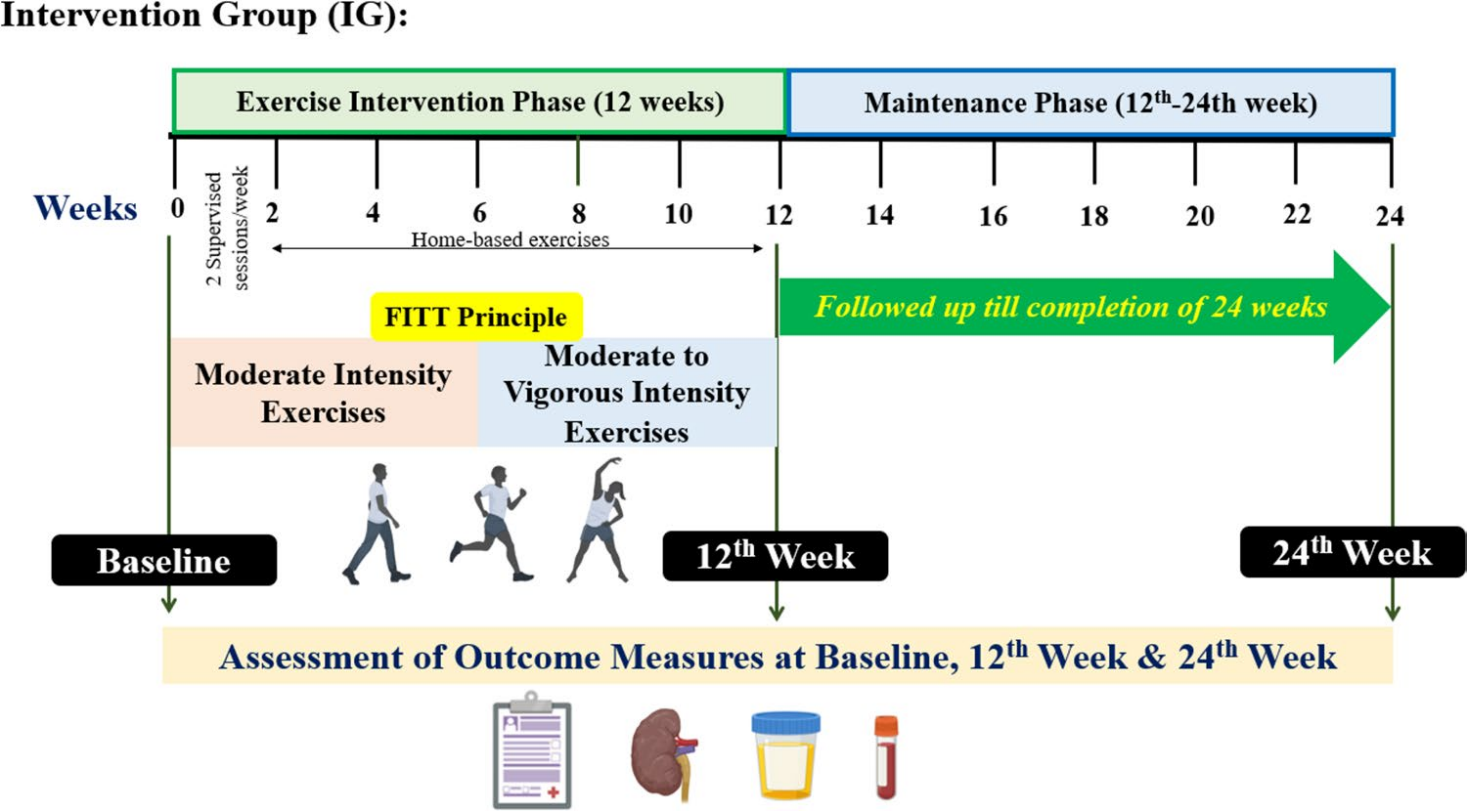
The exercise-based rehabilitation programme delivered to participants in intervention groups

At baseline, the detailed medical history, renal function outcome measurement, functional capacity assessment using 6-min walk tests were done. The exercise intervention was designed in accordance with the Template for Intervention Description & Replication (TIDieR) checklist to ensure the adequate delivery of exercise intervention was met in the study [17]. Furthermore, the authors utilized the “FITT principle” to describe the dosage of exercise in terms of the frequency (F), intensity (I), time/duration (T) and type of exercises to be performed (T) by the study participants. Participants were explained about selection of optimal intensity to exercise based on the rate of perceived exertion (RPE) using the original Borg scale, to help identify their fatigue level and thus slow down the intensity or stop and rest as required [18, 19]. They were explained about the benefits of engaging in regular exercises for the improvement of their overall health and kidney function. The structured exercises comprised of warm up (5 min), walking for aerobic activity (30–45 min), stretching (5 min) and cool-down (5 min) on 3–5 days/week. The body weight based exercises were performed on 2–3 consecutive days/week and targeted the major muscle groups of upper and lower extremity and trunk. The physiotherapists supervised two exercise sessions during the 1st and 2nd week after which participants continued with home-based exercises till completion of 12 weeks as depicted in Fig. 2**.**

#### Pre-requisite of exercise professional

The exercises were taught by a qualified cardio-pulmonary physiotherapist who was trained and certified to deliver both basic and advanced life support by the American Heart Association (AHA), in the event of any un-due medical emergency. Furthermore, the exercise professional was competent to immediately address any musculoskeletal injury, or episodes of hypo/hyperglycemia, dehydration, if experienced by the participants and ensure their safety during their exercise sessions.

#### Exercise adherence

Exercise Activity Log was provided to participants to record their engagement in the exercise sessions. They were reviewed by the physiotherapist during follow-up visits to the study center. The study team considered 70% as a valid score to define the exercise adherence of the participants (i.e., 42/60 sessions to be completed in 12 weeks of exercise intervention phase and similarly during maintenance phase till 24th week completion). Additionally, the exercise adherence rating scale (EARS) was also used to evaluate adherence of participants at the end of 12th and 24th week, respectively. EARS is 6 question-based scale where each question is rated on a 5 point Likert scale (0–4) with scores ranging between 0 and 24, with higher scores indicative of better adherence [20].

#### Follow-up

The study participants were followed up throughout the study period. Their health status was checked regularly through telephonic calls and text message reminders by the physiotherapist. They were provided with instructions for safe participation of exercise along with safety precautions.

## Statistical analysis

The study outcomes were recorded at three timepoints: baseline, post 12 weeks, post 24 weeks for both the groups (CG and IG). The baseline characteristics of study participants were reported using mean and standard deviation for continuous variables with normal distribution, or median and interquartile range for non-normally distributed data and absolute frequency and percentage for categorical variables. The Shapiro–Wilk p test was used to verify the normality of the outcome measures. The differences in the mean scores of the dependent variables were compared within each group by the repeated measures ANOVA across three timepoints. The final analysis comprised of 27 participants in CG and 28 in IG. The assumptions of the repeated measures ANOVA were checked using sphericity tests. In the event of violation of assumption, the Greenhouse–Geisser correction were performed for outcomes wherever deemed necessary. The statistical analysis for the study were performed using the Jamovi project (2022). *Jamovi.* (Version 2.3) [Computer Software]. https://www.jamovi.org. Significance was defined as *p* < 0.05. [21–24]

## Results

### Baseline and sociodemographic characteristics of study participants

A total of 283 individuals were screened, of which 223 were excluded due to several reasons as depicted in the CONSORT Flow Diagram Fig. 1. Among the screened individuals, a total of 60 eligible participants met the inclusion criteria and were explained about the study, and thereafter, a written informed consent was obtained from them prior to study participation.

The average age of the included study participants were 58.6 years (male = 52; female = 8). The average duration of type 2 diabetes mellitus among the participants were 11 years. The glycemic management for the participants were as follows: Diet + Oral hypoglycemic agent (OHAs) in 83.3%, followed by Diet + OHA + Insulin therapy in 10% and Diet + Insulin therapy in the remaining 6.7%. Hypertension was reported in all participants, and they were on medication for the same.

All participants were following a salt restriction in their diet as recommended to them by the dietetics and nutrition expert during their hospital visit. 66.7% of participants followed a vegetarian diet and 33.3% followed a non-vegetarian diet (comprising of meat, chicken, egg, and seafood). None of the participants were on any vitamin D supplements or reported a previous history of vitamin D deficiency. The addictions reported by participants were as follows: smoking = 8.3%, alcohol consumption = 20%, tobacco chewing = 1.7% only. Family history of diabetic kidney disease/chronic kidney disease were reported in only 8.5% (6 out of 60 included participants).

Baseline assessments of the primary and secondary outcome measures were performed for all the study participants followed by their random allocation to: CG-Control Group (*N* = 30 participants) and IG-Intervention Group (*N* = 30 participants). The baseline characteristics are described in Table 1. The two groups were comparable for all demographics (all *p* > 0.05).

**Table 1:** Baseline characteristics of study participants in both groups

Variable	Control group (CG) *N* = 30		Intervention group (IG) *N* = 30	
	Mean ± SD	Shapiro Wilk *p* value	Mean ± SD	Shapiro Wilk *p* value
Age (years)	58.3 ± 7.78	0.046	59 ± 7.60	0.021
BMI (kg/m^2^)	26.2 ± 3.63	0.061	26.2 ± 4.65	0.053
FBG (mg/dL)	146 ± 45.3	< 0.001	150 ± 42.0	0.322
PPBG (mg/dL)	233 ± 40.7	0.011	233 ± 51.4	0.004
HbA1c (%)	8.18 ± 1.98	< 0.001	8.27 ± 2.04	< 0.001
SBP (mm Hg)	137 ± 9.05	0.004	138 ± 9.97	0.184
DBP (mm Hg)	84.2 ± 7.26	< 0.001	83.9 ± 8.83	0.081
Baseline 6MWD (meters)	444 ± 25.1	0.122	445 ± 25.6	0.037
Serum Creatinine (mg/dL)	1.26 ± 0.53	< 0.001	1.18 ± 0.50	0.049
eGFR (mL/min/1.73m^2^)	73.2 ± 26.7	0.006	74.7 ± 27.6	0.034
Serum Urea (mg/dL)	26.1 ± 8.52	0.122	24.6 ± 10.9	0.047
Urine Protein (mg/dL)	23.17 ± 8.84	< 0.001	24.17 ± 8.84	< 0.001

*BMI* body mass index, *FBG* fasting blood glucose, *PPBG* post prandial blood glucose, *HbA1c* glycated hemoglobin, *SBP* systolic blood pressure, *DBP* diastolic blood pressure, *PR* pulse rate, *HR* heart rate, *SPO2* saturation of oxygen, *ABI* ankle brachial index, *eGFR* estimated glomerular filtration rate, *6MWD* six minute walk distance

### Changes in functional capacity

**Fig. 3:**
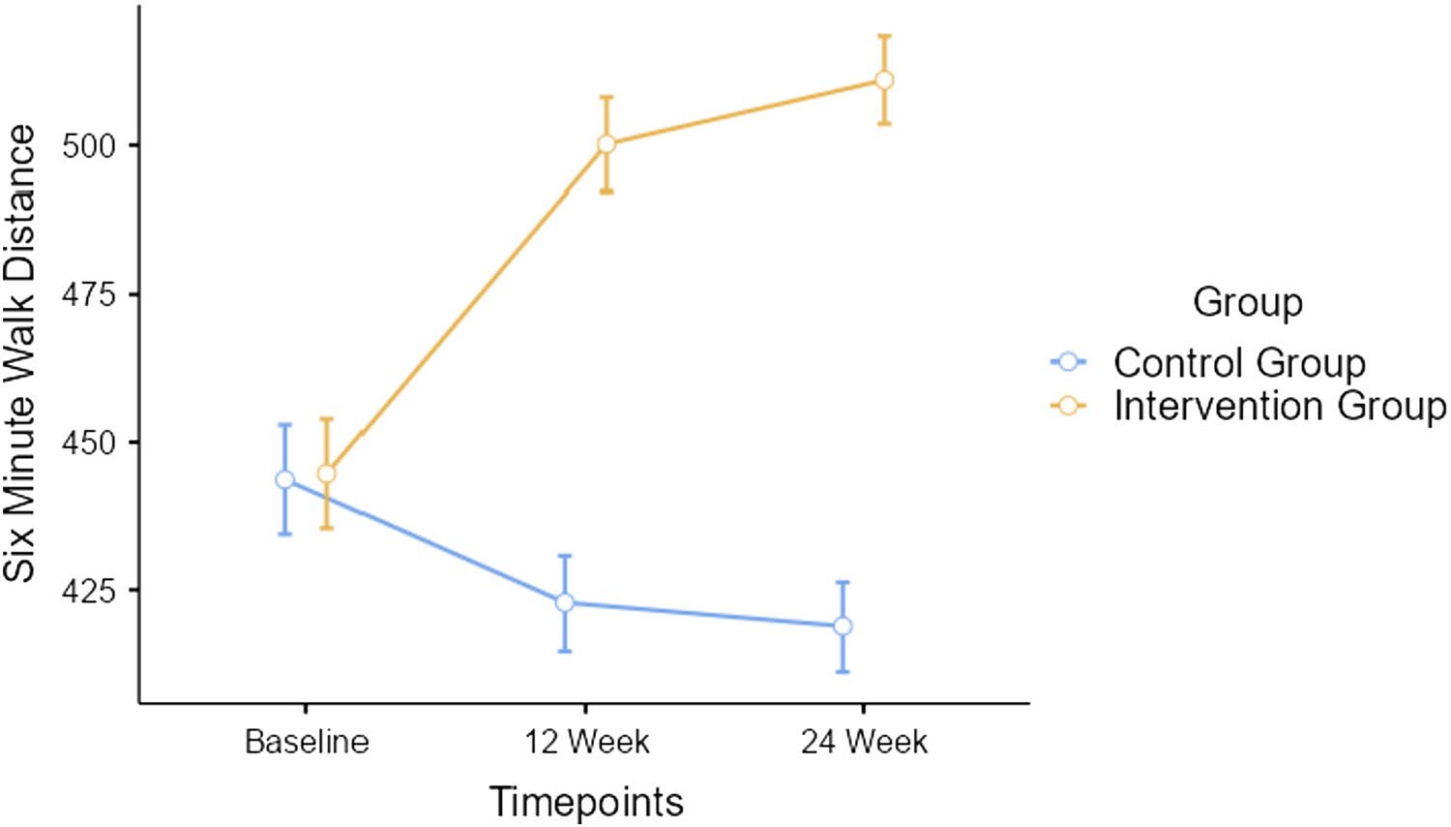
Changes in functional capacity—six minute walk distance between participants of both groups

Repeated measures ANOVA indicated significant timepoint*group interaction effect for six minute walk distance outcome with Greenhouse–Geisser corrected *F*_(1.71, 90.59)_ = 619, *p* < 0.001, depicted in Fig. 3.

### Changes in renal function

**Fig. 4:**
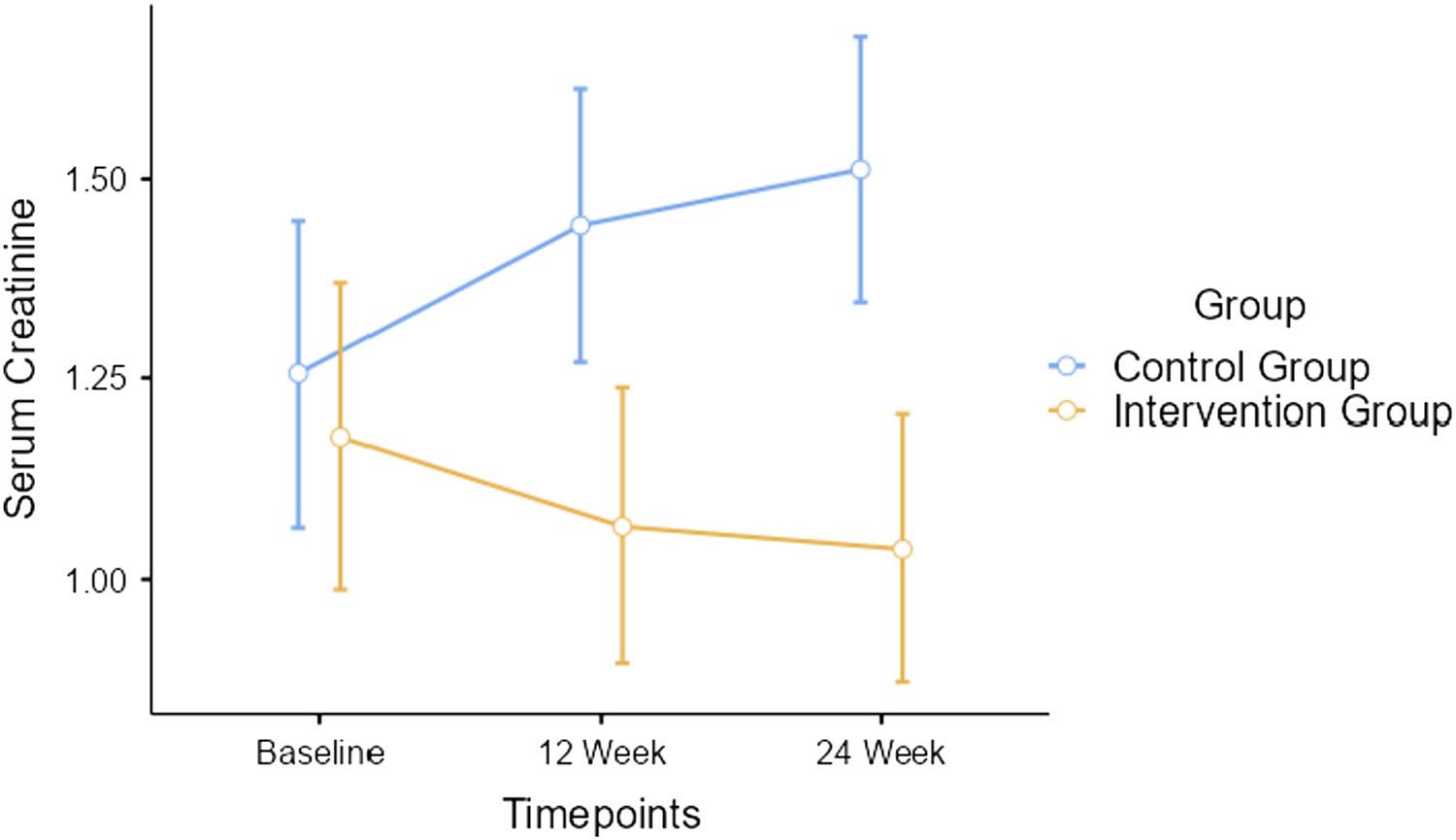
Changes in renal function—serum creatinine between participants of both groups

**Fig. 5:**
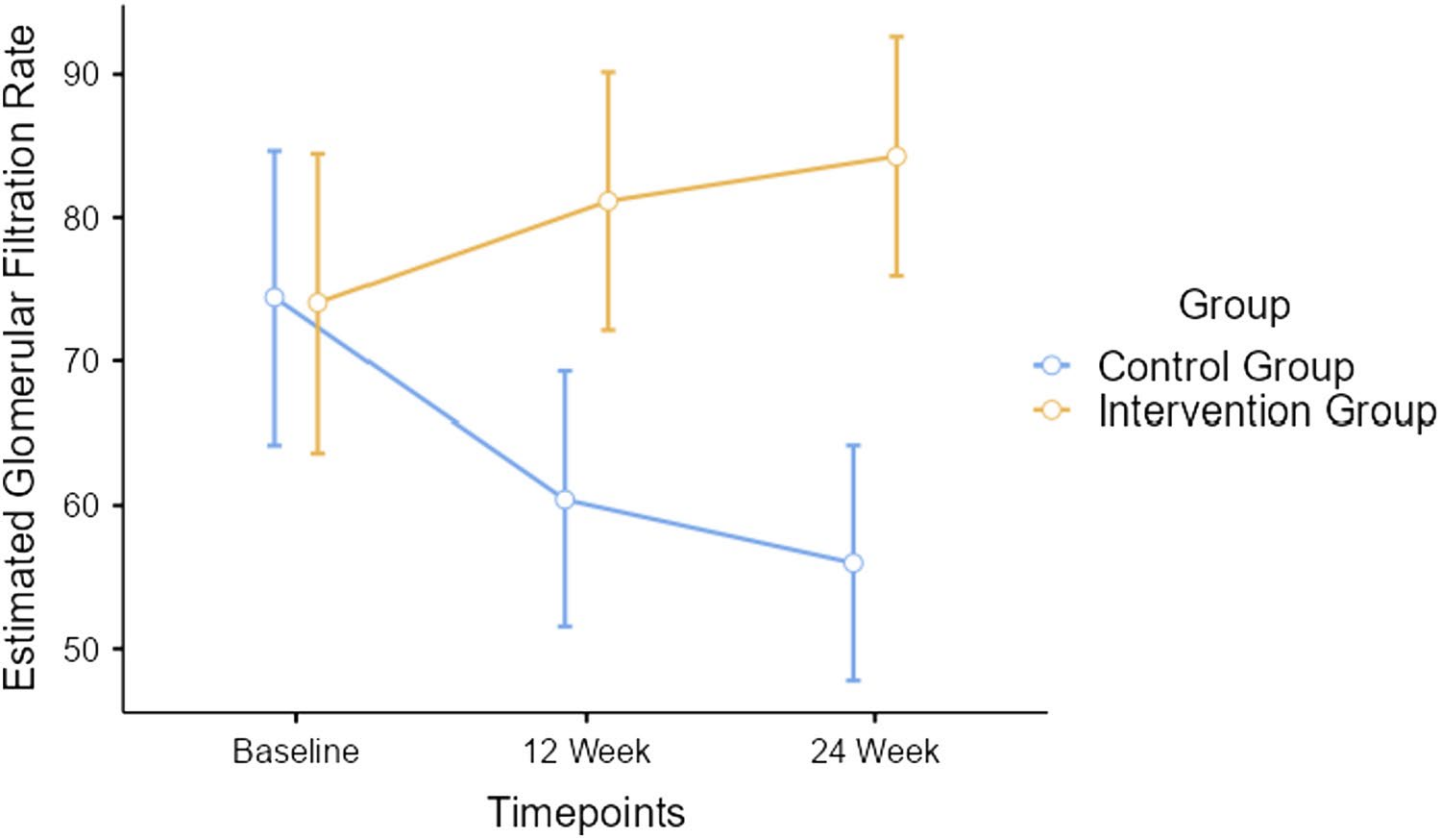
Changes in renal function—estimated glomerular filtration rate between participants of both groups

**Fig. 6:**
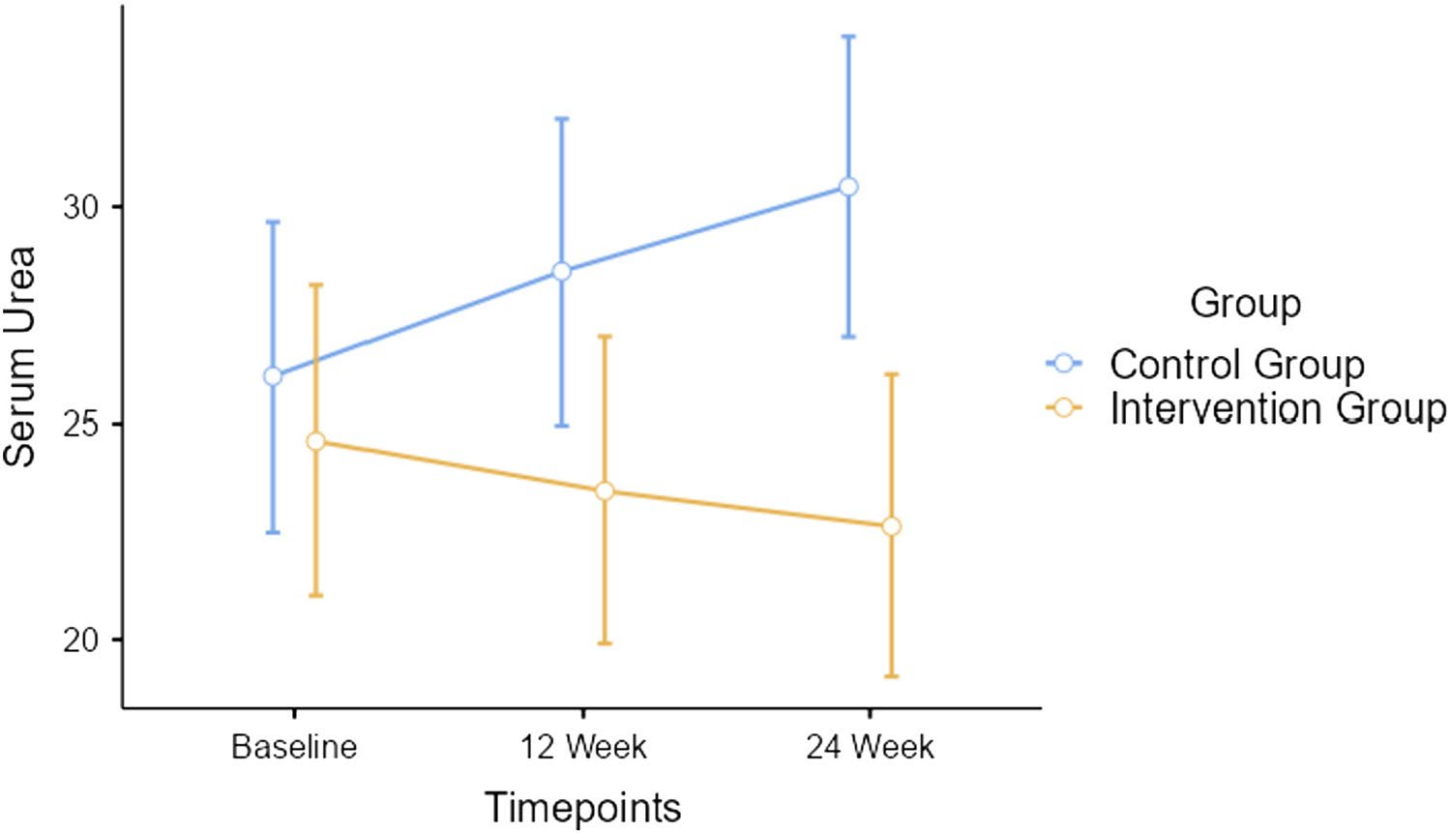
Changes in renal function—serum urea between participants of both groups

**Fig. 7:**
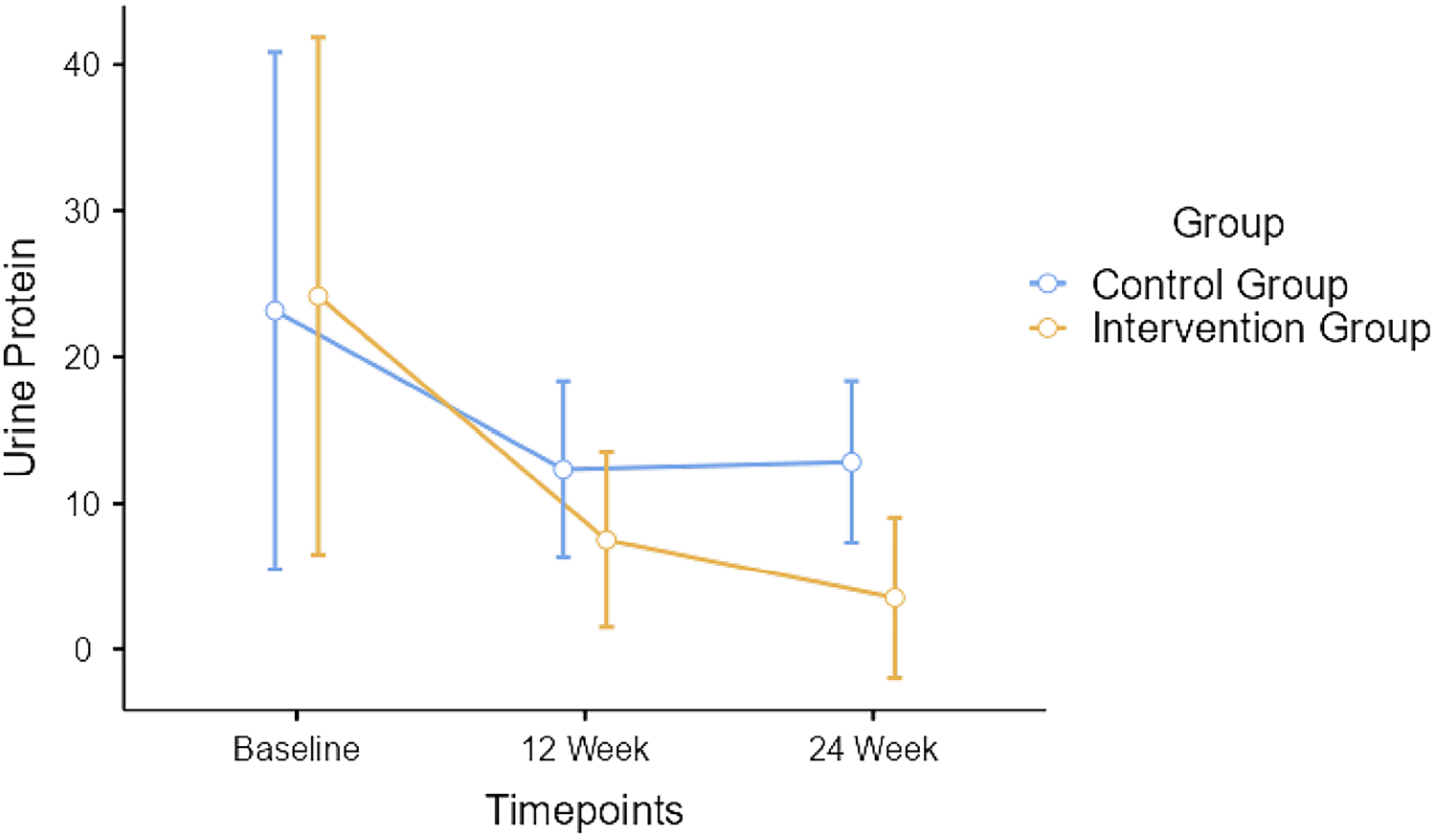
Changes in renal function—urine protein level between participants of both groups

Repeated measures ANOVA indicated significant timepoint *group interaction effect for serum creatinine outcome measure with Greenhouse–Geisser corrected *F*_(1.23, 65.14)_ = 174.8, *p* < 0.001, depicted in Fig. 4. Repeated measures ANOVA indicated significant timepoint*group interaction effect for eGFR with Greenhouse–Geisser corrected *F*_(1.15, 60.88)_ = 105.2, *p* < 0.001, depicted in Fig. 5. Repeated measures ANOVA indicated significant timepoint*group interaction effect for serum urea with Greenhouse–Geisser corrected *F*_(1.48, 78.45)_ = 261.4, *p* < 0.001, depicted in Fig. 6. Repeated measures ANOVA indicated significant timepoint*group interaction for urine protein with Greenhouse–Geisser corrected *F*_(1.13, 59.82)_ = 4.58, *p* < 0.328, depicted in Fig. 7.

## Discussion

The randomized controlled study aimed to determine the effect of exercise-based rehabilitation on functional capacity and renal function among individuals with type 2 diabetic nephropathy. The study found a significant difference between functional capacity and renal function outcome measures among intervention group participants as compared to control group. Interestingly, on comparing the slopes of function capacity and renal function outcome measures, the intervention group participants showed significant improvement when compared to controls. The exercise program had sustained improvement on the outcomes between 12 and 24th week of intervention group as compared to controls. This is indicative that the exercise program was feasible, and participants continued to engage in exercise even during their maintenance phase. They met the required adherence set by the study team. Given the alarmingly high probability of development of renal function decline among type 2 diabetes mellitus individuals, a sustainable approach to encourage & recommend adoption of exercises in routine therapy has emerged as a strong necessity to maintain health and functional capacity (Table 2).

**Table 2:** Estimated marginal means for functional capacity & renal function outcome measures of participants in both groups across timepoints

Outcome measures	Group	Timepoints	Mean	SE	95% Confidence interval	
					Lower	Upper
Six-minute walk Distance (meters)	Control group	Baseline	446	4.93	436	455
		12 Week	424	4.16	416	432
		24 Week	419	3.93	412	427
	Intervention group	Baseline	444	4.84	434	454
		12 Week	499	4.08	491	507
		24 Week	511	3.86	503	518
Serum Creatinine (mg/dL)	Control group	Baseline	1.17	0.09	0.98	1.36
		12 Week	1.37	0.08	1.19	1.54
		24 Week	1.45	0.08	1.28	1.62
	Intervention group	Baseline	1.21	0.09	1.02	1.40
		12 Week	1.09	0.08	0.92	1.26
		24 Week	1.06	0.08	0.89	1.22
Estimated Glomerular Filtration Rate (mL/min/1.73m^2^)	Control group	Baseline	78.2	5.25	67.7	88.7
		12 Week	62.9	4.64	53.6	72.2
		24 Week	58.0	4.33	49.3	66.6
	Intervention group	Baseline	73.1	5.15	62.7	83.4
		12 Week	80.3	4.56	71.2	89.5
		24 Week	83.6	4.25	75.1	92.1
Serum Urea (mg/dL)	Control group	Baseline	25.1	1.90	21.3	29.0
		12 Week	27.6	1.87	23.8	31.4
		24 Week	29.8	1.86	26.0	33.5
	Intervention group	Baseline	25.1	1.86	21.4	28.9
		12 Week	24.0	1.84	20.3	27.7
		24 Week	23.1	1.83	19.4	26.8
Urine Protein (mg/dL)	Control group	Baseline	14.63	5.89	2.80	26.45
		12 Week	10.00	2.21	5.57	14.43
		24 Week	10.56	1.76	7.02	14.09
	Intervention group	Baseline	22.32	5.79	10.71	33.93
		12 Week	6.96	2.17	2.61	11.31
		24 Week	2.68	1.73	-0.79	6.15

Our study addresses this concept in the Indian setting. From baseline till 24 weeks, functional capacity improved by 16.3% in intervention group and decreased by 6.05% in control group. This is an important finding from our study. A previous study by Kohl et al. 2012 reported that the survival of end-stage renal disease (ESRD) patients increased by ≈ 5% for every 100 m walked in 6-min walk test [25]. Thus, the regular clinical evaluation of functional capacity in hospital/clinic/private settings may aid in the early identification of diabetic nephropathy individuals at a risk for developing physical health deterioration in the months that follow. Any change in the functional capacity will enable healthcare providers to plan and promptly execute exercise-based rehabilitation for individuals with diabetic nephropathy.

After 12 weeks, the serum creatinine level of participants in intervention group were maintained within the normal range, while it increased by 0.2 mg/dL among control group. After 24 weeks, they were maintained for the intervention group but increased by 0.08 mg/dL among the control group. We found a significant increase in eGFR by 7.2 mL/min per 1.73 m^2^ after 12 weeks among intervention group participants and decreased of 15.3 mL/min per 1.73 m^2^ in control group. Moreover, the eGFR further improved after completion of 24 weeks by 3.3 mL/min per 1.73 m^2^ indicative of a sustained effect of the intervention during maintenance phase for the intervention group participants while it decreased by 4.9 mL/min per 1.73 m^2^ among controls. This is suggestive of a potential effect of exercise on renal function. The authors have explored the knowledge and identified the gaps in clinical nephrology literature through their systematic review and meta-analysis [11].

Serum urea decreased by 1.1 mg/dL after 12 weeks among intervention group participants and increased by 2.5 mg/dL among control group. After 24 weeks, serum urea decreased by 0.9 mg/dL among intervention group participants and increased by 2.2 mg/dL for the control group. However, the changes in serum urea were well-within the normative range. The urine protein level decreased by 15.36 mg/dL after 12 weeks among intervention group participants and decreased by only 4.6 mg/dL for the control group. While, after 24 weeks, urine protein decreased by 4.28 mg/dL among intervention group participants and increased by 0.5 mg/dL for the control group. The decrease in urine protein levels were better observed in intervention group as compared to control group participants.

The maintenance of hemodynamic balance is a key function of the kidney while an optimal renal blood pressure is an indicator of its vascular stability. Individuals with diabetes are susceptible to blood pressure elevations due to impaired renal-autoregulatory mechanisms. The diabetic milieu increases the weight and size of the kidney by an average of 15% due to glomerular hyperfiltration, renal hypertrophy and altered glomerular composition. Long-term chronic hyperglycemia and hypertension among individuals with type 2 diabetic nephropathy manifests clinically as proteinuria, albuminuria or altered glomerular filtration rate, etc. The present study utilized Borg RPE for selection and quantification of exercise intensity for prescription as it supports patient autonomy. Borg RPE is a simple, safe method which saves time of both participant and exercise therapist, while allowing ease in follow-up and exercise progression over simple telephonic mode [19]. Body weight-based exercises enhance activity of skeletal muscle, which is a metabolically active tissue of the body. An increase in skeletal muscle mass equals availability of more creatinine storage within them [26]. Thus, limiting amount of freely circulating creatinine in the bloodstream (serum creatinine). Moreover, maintenance of serum creatinine in normal range equals stabilization of eGFR level. A metabolically deranged micro-environment also leads to over-production of nitrogenous wastes in the blood.

Most nephrology clinical trials have targeted ESRD or dialysis or renal transplant population at large with very few randomized controlled trials focusing on the diabetic nephropathy population [27]. Existing pilot study or clinical trials on various stages of chronic kidney disease/diabetic kidney disease/type 2 diabetic nephropathy lack large sample size or long-term follow-up or fail to discuss the maintenance effect of the exercise intervention on renal function. Concerns about safety, exercise adherence, long-term behaviour change among diabetic nephropathy population demands future research.

This is a single large randomized controlled trial evaluating the effect of exercise on functional capacity and renal function among type 2 diabetes mellitus individuals with nephropathy. The study had one limitation—the engagement of physical activity and adherence to diet among control group participants were not recorded by the study team. This could have impacted the decline in the study outcome measures among control group participants. Future studies must evaluate the long-term effects of the exercise intervention up to 1 year and beyond, for different stages of nephropathy. Furthermore, the improvements in skeletal muscle due to the study intervention may be evaluated in future studies using objective measures.

## Conclusion

The study found that exercise-based rehabilitation program improved the functional capacity and renal function among individuals with type 2 diabetic nephropathy.

## Data Availability

The study includes participant data which will be made available based on reasonable request to the corresponding author.
